# Sex-Specific Heritability of Spontaneous Lipid Levels in an Extended Pedigree of Indian-Origin Rhesus Macaques (*Macaca mulatta*)

**DOI:** 10.1371/journal.pone.0072241

**Published:** 2013-08-08

**Authors:** Amanda Vinson, Asia D. Mitchell, David Toffey, Jacob Silver, Michael J. Raboin

**Affiliations:** 1 Oregon National Primate Research Center, Beaverton, Oregon, United States of America; 2 Department of Molecular and Medical Genetics, Oregon Health & Science University, Portland, Oregon, United States of America; 3 Oregon National Primate Research Center Summer Undergraduate Fellowship Program, Beaverton, Oregon, United States of America; Tulane University, United States of America

## Abstract

The rhesus macaque is an important model for human atherosclerosis but genetic determinants of relevant phenotypes have not yet been investigated in this species. Because lipid levels are well-established and heritable risk factors for human atherosclerosis, our goal was to assess the heritability of lipoprotein cholesterol and triglyceride levels in a single, extended pedigree of 1,289 Indian-origin rhesus macaques. Additionally, because increasing evidence supports sex differences in the genetic architecture of lipid levels and lipid metabolism in humans and macaques, we also explored sex-specific heritability for all lipid measures investigated in this study. Using standard methods, we measured lipoprotein cholesterol and triglyceride levels from fasted plasma in a sample of 193 pedigreed rhesus macaques selected for membership in large, paternal half-sib cohorts, and maintained on a low-fat, low cholesterol chow diet. Employing a variance components approach, we found moderate heritability for total cholesterol (h^2^=0.257, P=0.032), LDL cholesterol (h^2^=0.252, P=0.030), and triglyceride levels (h^2^=0.197, P=0.034) in the full sample. However, stratification by sex (N=68 males, N=125 females) revealed substantial sex-specific heritability for total cholesterol (0.644, P=0.004, females only), HDL cholesterol (0.843, P=0.0008, females only), VLDL cholesterol (0.482, P=0.018, males only), and triglyceride levels (0.705, P=0.001, males only) that was obscured or absent when sexes were combined in the full sample. We conclude that genes contribute to spontaneous variation in circulating lipid levels in the Indian-origin rhesus macaque in a sex-specific manner, and that the rhesus macaque is likely to be a valuable model for sex-specific genetic effects on lipid risk factors for human atherosclerosis. These findings are a first-ever report of heritability for cholesterol levels in this species, and support the need for expanded analysis of these traits in this population.

## Introduction

The rhesus macaque has a lengthy history as a non-human primate (NHP) model for the physiology of atherosclerosis, particularly in response to experimental diets rich in fat and cholesterol [1-9]. Depending on the type and amount of dietary fat and cholesterol, and the length of time fed, rhesus macaques develop either mild hypercholesterolemia similar to that commonly seen in the general human population, or the more extreme hypercholesterolemia seen in familial human disease [2,3,5,8,9]. Coincident with this increase in plasma cholesterol and in direct proportion to the length of time on the experimental diet, rhesus macaques develop increasingly severe atherosclerosis that is strikingly similar to that found in humans. Observed stages of lesion development include the retention of lipid-rich macrophages (foam cells) and other immune cells in the subendothelium, the appearance of fatty streaks in the aorta and coronary arteries, the progression from fatty streak to uncomplicated fibrous atheroma with proliferation of smooth muscle cells, and eventually to more complicated lesions characterized by intimal necrosis, calcification, and hemorrhage [4,5,7]. Of particular note, rhesus macaques fed experimental diets also progress to clinically relevant stages of disease [1], including ischemia with significant stenosis of the coronary arteries, occlusive thrombosis, myocardial infarction, and sudden death [5].

In contrast to its success as a physiological model for atherosclerosis, the use of the rhesus macaque to discover genetic determinants of atherosclerosis is currently nonexistent, primarily due to a lack of genotyped and phenotyped study populations. This is unfortunate, given the well-established role of genetics in human atherosclerosis [10,11] and related inflammation [12,13], the close genetic similarity between macaques and humans, the feasibility of developing large pedigrees of macaques in managed settings like primate research centers, and the importance of this species as a pre-clinical model for human therapeutics. Studies of complex disease traits typically require very large sample sizes to have sufficient power for genetic discovery; however, an extended pedigree of rhesus macaques may have greater analytical power than a population-based study cohort of the same size, and moreover will be enriched for low-frequency genetic variants, which are increasingly implicated in susceptibility to disease [14]. Additionally, because animals experience reduced environmental heterogeneity in a managed setting (e.g., the same diet, housing, veterinary care, etc.), power to detect genetic signal over noise is further enhanced.

Here, we describe an important first step toward identifying genetic determinants of atherosclerosis in the rhesus macaque by characterizing the contribution of genes (i.e., heritability) to spontaneous variation in circulating lipids. We elected to study lipid levels first because lipids are well-established risk factors for human atherosclerosis, and because heritability for lipid levels has been demonstrated previously in both humans and in other NHP species [15,16]. Additionally, because significant gender differences in the genetic architecture of lipid levels and lipid metabolism have been demonstrated recently in humans [17-19], and implicated in rhesus macaques [20], we also wanted to investigate potential differences in heritability between male and female macaques for all lipids measured in this study. Thus, the aims of this study were to develop a single, extended pedigree of Indian-origin rhesus macaques with sufficient power for quantitative trait analysis, and to assess total and sex-specific heritability for baseline lipoprotein cholesterol and triglyceride levels in a sample of pedigreed macaques fed a standard chow diet low in fat and cholesterol.

## Methods

### Ethics statement

The majority of animals in this study were maintained outdoors in free-ranging social groups of approximately 120-170 individuals within 1-acre enclosures containing climbing and play structures. Indoor-housed monkeys were maintained as pairs (i.e., two individuals occupy two or more cages to which they both have full access) when possible; singly-housed monkeys were maintained in visual, auditory, and olfactory contact with conspecifics. Additionally, all monkeys participated in the Oregon National Primate Research Center (ONPRC) Behavioral Management, Plan; this Plan provides daily foraging opportunities, toys and other manipulatable objects that are rotated regularly, and additional access to other enrichment including television and radio (for indoor-housed animals). Animal care personnel and staff veterinarians of the ONPRC provide routine and emergency health care to all animals in accordance with the Guide for the Care and Use of Laboratory Animals. The ONPRC is certified by the Association for Assessment and Accreditation of Laboratory Animal Care International, and all procedures were approved by the Institutional Animal Care and Use Committee of the Oregon Health & Science University (Protocol Number: IS00002621). All efforts were made to minimize animal stress and discomfort during sampling procedures.

### Pedigree characterization and sample biobanking

We aimed to develop a single, extended pedigree of Indian-origin rhesus macaques (*Macaca mulatta*) to enable genome-wide genetic analysis of quantitative risk factors for atherosclerosis and related complex human disease. This pedigree was characterized by developing custom computational scripts to select an optimal set of animals from the ~4,500-member colony of rhesus macaques housed at the ONPRC based on several criteria: animals must, 1) be of pure Indian ancestry; 2) have parentage assignment based on genotypes at 12-28 microsatellite loci; 3) be a member of a minimum 3-generation vertical lineage; 4) be available for sampling; 5) have no ancestors in common outside the pedigree; and 6) form a single pedigree structure.

To enable extensive phenotyping of pedigree members, we collected whole blood samples on these macaques under an approved Institutional Animal Care and Use Committee (IACUC) protocol. In general, samples were collected during routine sedation for veterinary care, following an overnight fast and at a consistent time of day (~10: 00 a.m.). Twenty (20) mLs whole blood was collected into EDTA, heparin, or no preservative, as indicated, during a standard 15-20 minute sedation by intramuscular injection of ketamine or telazol, performed by technical personnel in the Div. of Comparative Medicine at ONPRC. Whole blood was processed for serum, plasma, leukocytes, and peripheral blood mononuclear cells (PBMCs), aliquoted separately for storage in RNAlater® (Ambion) to enable future gene expression analysis, and into FBS/DMSO for storage in liquid nitrogen to enable viable cell assays. Samples are maintained at -80° C, except where otherwise indicated; for maximum efficiency, serum and plasma is stored in 0.1 mL single-use aliquots.

### Focal animal set

Because we plan to measure multiple phenotypes related to both lipids and inflammation in this initial sample of macaques, we minimized the potential influence of co-morbid medical conditions on heritability by preferentially selecting animals with banked samples according to the following criteria. No animals which were part of experimental research procedures at the time of sampling were allowed. Additionally, animals with the following terms in their medical records within the 6 months prior to sampling were not considered for analysis: “arthritis”, “diarrhea”, “chronic”, “cancer”, “tumor”, “hepatomegaly”, and “liver enlargement”, or “trauma” within the 2 weeks prior to sampling. Animals passing this medical screen were then preferentially selected according to membership in large paternal half-sibship cohorts and sample availability. Ultimately, our focal sample included 193 pedigreed macaques comprised of 125 females and 68 males, ranging in age from 1.9–21.6 years, corresponding approximately to a human developmental age range of 5.7–64.8 years. All animals were maintained on a standard commercial low-fat monkey chow diet (LabDiet® Lab Fiber-Balanced Monkey Diet, 14.7% fat, 27 ppm cholesterol, or LabDiet® High Protein Monkey Diet, 13.2% fat, 70 ppm cholesterol, PMI Nutrition International, Brentwood, MO) to which they have *ad libitum* access. Monkeys were offered food in excess in order to ensure that all animals have unconstrained access to food irrespective of individual social dominance rank, which may confound heritability analysis when food is limiting.

### Cholesterol assays

Analyses of total cholesterol (TCHOL), LDL cholesterol (LDL-C), HDL cholesterol (HDL-C), VLDL cholesterol (VLDL-C), and triglyceride (TRIG) levels were performed by the Oregon Health & Science University Lipid Laboratory on EDTA plasma collected and stored as described above. Levels of total cholesterol and triglycerides were measured by enzymatic colorimetric methods using Cobas® reagents Cholesterol CHOD-PAP and Triglycerides GPO-PAP (Roche Diagnostics, Indianapolis, IN), respectively. Inter-assay CV is <2.0% for total cholesterol, and <5.0% for triglycerides. HDL cholesterol (HDL-C) levels were measured with a homogeneous enzymatic colorimetric test, using Cobas® HDL-C Plus 3^rd^ Generation, with an inter-assay CV <3.5%. All analyses were performed using a Hitachi 704 Chemistry Analyzer. LDL- and VLDL cholesterol levels were calculated using the Friedewald equation [21].

### Quantitative genetic analysis

The goal of a quantitative genetic analysis is to partition total trait variance () into additive genetic () and environmental () components. To achieve this, we used a maximum likelihood-based variance decomposition approach implemented in the software package SOLAR (Sequential Oligogenic Linkage Analysis Routines). This approach, described in detail elsewhere [22], partitions the observed phenotypic covariance in each trait into components corresponding to additive genetic and environmental effects as, where is the matrix of phenotypic (co) variance among individuals, is a matrix of expected kinship coefficients among relative pairs, is an identity matrix that implies an unshared environment unique to each individual, and and are the additive genetic and environmental variances being estimated [23]. We modeled phenotypes as*y*=*μ*+*β*_1_ *x*_1_+*β*_2_ *x*_2_+....+*β*_*n*_ *x*_*n*_+*g*+*e*, in which μ is the trait mean in the population, *β*_*i*_ are the coefficients for covariate effects on the mean, *x*_*i*_ are covariate values, and *g* and *e* are genetic and environmental effects, respectively. Covariate-specific trait means are used to calculate the phenotypic covariance among relative pairs [24], and evaluation of the phenotypic covariance matrix allows estimation of the additive genetic and environmental variance components. Heritability is then estimated as the proportion of residual phenotypic variance unexplained by covariates that can be attributed to additive genetic effects (i.e., h^2^ =), and the environmental contribution to residual phenotypic variance is estimated as *e* ^2^ = 1−*h* ^2^.

Parameter estimates were obtained by the method of maximum likelihood, with statistical significance assessed using likelihood ratio (LR) tests in which the likelihood of a general model containing the parameter estimate was compared to the likelihood of a restricted model in which the parameter was constrained to be zero. Degrees of freedom in these tests are equal to the difference in the number of parameters estimated in the two models. In the case where zero does not lie on the boundary of parameter space (e.g., in the restricted model for LR tests of covariates), the likelihood ratio statistic (equal to twice the difference in the ln likelihoods of the two models) is distributed as, and unadjusted P-values are used. For LR tests in which zero is at the boundary of parameter space (e.g., heritability), the LR statistic is distributed asymptotically as a ½: ½ mixture of and a point mass at zero, requiring an adjusted significance level equal to one-half the P-value obtained for [25,26].

All data obtained was used in this study. However, raw data for both VLDL-C and triglyceride levels displayed an unacceptable degree of leptokurtosis. To correct this, we applied an inverse Gaussian transform to all lipid data to ensure both normality and consistency in our approach. The statistical genetic goals of this study were to assess the additive genetic contributions to variation in lipids, 1) exclusive of the effects of age and sex, and 2) exclusive of age within the environment of sex. To estimate the heritability of lipid levels exclusive of age and sex effects, we assessed the effects of covariates age, sex, age × sex, age^2^, and age^2^ × sex on the estimate of heritability, and the set of covariates that produced the maximum estimate of heritability was retained in the final model. To estimate sex-specific heritability of lipid levels, we stratified the sample according to sex and followed the same procedure, screening only for covariates age and age^2^.

## Results

### Pedigree

**Figure 1:**
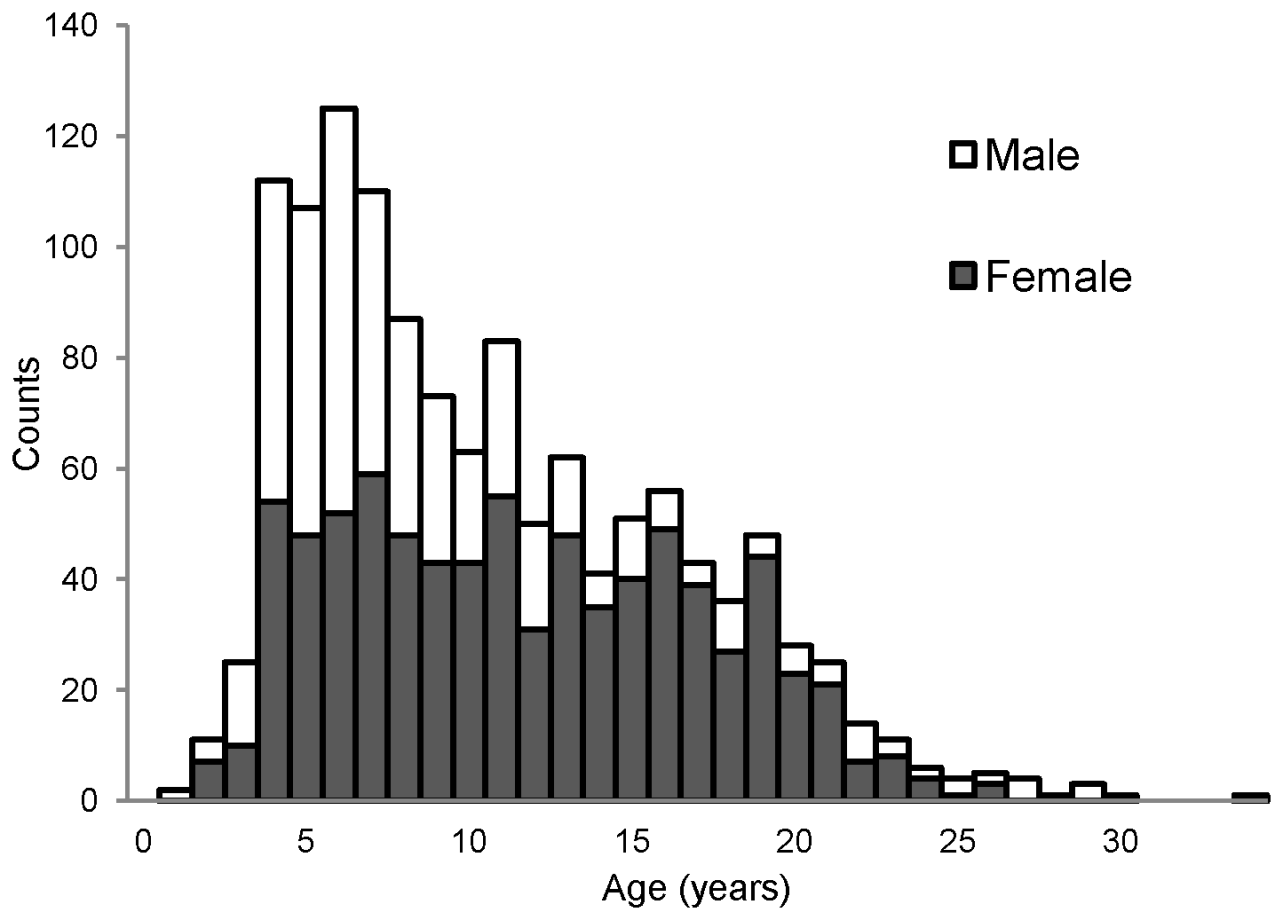
Frequency histogram of age by sex for all rhesus macaques in the single, 1,289-member pedigree described.

The resulting pedigree contains 1,289 rhesus macaques, including 800 females and 489 males. This pedigree represents approximately 29% of the total population of rhesus macaques found at the ONPRC. Animals range in age from 1.6–28.5 years, corresponding to a developmental age range of 4.8–85.5 human years (the distribution of age by sex in this pedigree is summarized in Figure **1**). We have banked blood components on >840 animals to date, and 1,251 (~97%) animals in this pedigree have at least an available source of high-quality genomic DNA for future genetic analysis.

TABLE **1** summarizes the most frequent relationship classes found within this pedigree. Power analyses indicate this single pedigree configuration has excellent power for variance component analysis and gene mapping using either linkage or association approaches (see 27 for an expanded discussion). For heritability analysis, this pedigree has ~85% power to detect heritability as low as 0.08. For linkage analysis, this pedigree has >80% power to detect QTL heritabilities as low as 0.15 with a LOD score of 3, assuming total trait heritability of 0.25 with all animals phenotyped. For association analysis using a measured genotype approach, this pedigree exhibits 80% power to detect association with a causal variant (or a marker SNP in complete linkage disequilibrium with the causal variant) accounting for between 1.5–2.0% of total variance in a representative quantitative trait, assuming total trait heritability of 20% and a minor allele frequency of 0.20.

**Table 1:** A summary of the most frequent relationship types found within the single, 6-generation 1,289-member pedigree described.

**RELATIONSHIP**	**NOS.**	**RELATIONSHIP**	**NOS.**
Unrelated	890,106	Half 2nd cousins	1,284
Parent-offspring	1,966	Half-siblings and half 1st cousins	371
Siblings	165	Half siblings & half avuncular	73
Grandparent-grandchild	2,150	Double half avuncular	193
Avuncular	222	Double half 1st cousins	358
Half-siblings	6,954	Half 2nd cousins, once removed	128
Great grandparent-grandchild	1,027	Half 1st cousins, twice removed	209
Grand avuncular	56	Half great grand avuncular	98
Half avuncular	13,179	Half 1st cousins & half avuncular	142
1st cousins	156	Half-avuncular and half-grand avuncular	30
Great great grandparent-grandchild	84	Half avuncular & half 1st cousins, once removed	330
Half grand avuncular	3,079	Half 1st cousins & half 2nd cousins	139
1st cousins, once removed	128	Parent-offspring & half avuncular	16
Half 1st cousins	10,413	Half 1st cousins, once removed & half 2nd cousins, once removed	14
Half 1st cousins, once removed	6,740	Half sibs & half 2nd cousins	24
2nd cousins	27		

### Distribution of observed lipid levels

**Figure 2:**
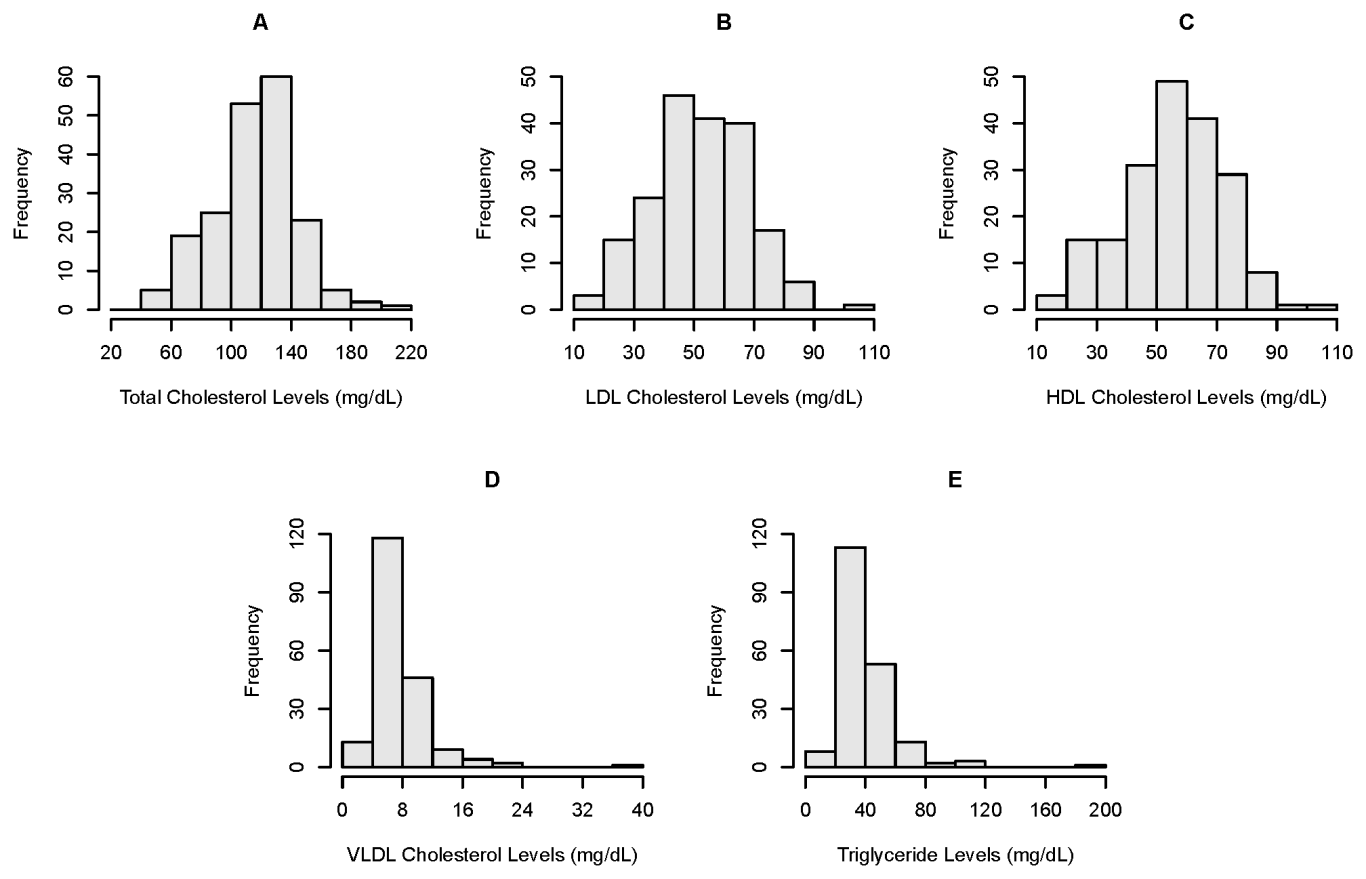
Frequency histogram of total, LDL-, HDL-, VLDLcholesterol and triglyceride levels in 193 rhesus macaques maintained on a low-fat, low-cholesterol diet.

Considering the basal, low-fat diet fed to these macaques, we found substantial variation among individual animals for all lipids assayed in this study (see Figure **2**). All descriptive data (TABLES **2**-**6**, Figure **2**) is presented untransformed for ease of interpretation. TABLES **2**-**6** summarize measures of centrality (mean and median) and variation for all untransformed data, calculated separately for the 68 males and 125 females, and for the sample as a whole (N=193). Means and median values for each lipid measure are similar, consistent with an underlying normal or approximately normal distribution for these traits in this population. Although mean values for all lipids were well within the recommended guidelines for human cholesterol levels, values for 6 animals (2 of the 6 are related as half-sibs, with the remainder unrelated) were observed for several lipids that were at or beyond 3 standard deviations from their respective sex-specific means. In a human clinical setting, these values would be considered higher than desirable for LDL-C, higher than normal for VLDL-C, and “borderline high” for total cholesterol and triglycerides [28].

**Table 2:** Distribution of total cholesterol levels in 193 pedigreed rhesus macaques maintained on low-fat, low-cholesterol chow diet (unadjusted data in mg/dL).

	**MALES (N=68)**	**FEMALES (N=125)**	**ALL (N=193)**
MEAN	124.00	113.52	117.21
MEDIAN	122.50	114.00	118.00
VARIANCE	462.63	907.91	772.99
LOW VALUE	71.00	49.00	49.00
HIGH VALUE	188.00	201.00	201.00

**Table 3:** Distribution of LDL cholesterol levels in 193 pedigreed rhesus macaques maintained on low-fat, low-cholesterol chow diet (unadjusted data in mg/dL).

	**MALES (N=68)**	**FEMALES (N=125)**	**ALL (N=193)**
MEAN	54.51	51.94	52.85
MEDIAN	54.00	50.00	53.00
VARIANCE	218.97	282.49	260.37
LOW VALUE	21.00	15.00	15.00
HIGH VALUE	90.00	101.00	101.00

**Table 4:** Distribution of HDL cholesterol levels in 193 pedigreed rhesus macaques maintained on low-fat, low-cholesterol chow diet (unadjusted data in mg/dL).

	**MALES (N=68)**	**FEMALES (N=125)**	**ALL (N=193)**
MEAN	60.76	53.54	56.09
MEDIAN	58.00	55.00	56.00
VARIANCE	166.51	317.93	275.39
LOW VALUE	39.00	13.00	13.00
HIGH VALUE	90.00	106.00	106.00

**Table 5:** Distribution of VLDL cholesterol levels in 193 pedigreed rhesus macaques maintained on low-fat, low-cholesterol chow diet (unadjusted data in mg/dL).

	**MALES (N=68)**	**FEMALES (N=125)**	**ALL (N=193)**
MEAN	8.72	7.87	8.17
MEDIAN	8.00	7.00	7.00
VARIANCE	12.20	16.26	14.92
LOW VALUE	4.00	3.00	3.00
HIGH VALUE	21.00	38.00	38.00

**Table 6:** Distribution of triglyceride levels in 193 pedigreed rhesus macaques maintained on low-fat, low-cholesterol chow diet (unadjusted data in mg/dL).

	**MALES (N=68)**	**FEMALES (N=125)**	**ALL (N=193)**
MEAN	42.87	39.50	40.68
MEDIAN	39.50	36.00	37.00
VARIANCE	270.42	400.09	355.36
LOW VALUE	19.00	17.00	17.00
HIGH VALUE	103.00	188.00	188.00

### Effects of sex and age on observed lipid levels

The mean and variance of all lipid levels investigated differed by age and by sex, and at least some corresponding covariate effects are statistically significant for each lipid (see significant covariates in TABLE **7**), accounting for up to ~19% of observed variation. Notably, covariate effects related to sex only, and/or interactions between sex and age, occurred in 3 of the 5 lipid measures. Consistent with strong effects of sex, mean and median values for all lipids investigated were higher in males than females. In contrast, variance in all lipid levels (both absolute value and as a proportion of the mean) was greater in females than in males. As a proportion of the mean, variance was 8.00 in females vs. 3.73 in males for total cholesterol; similarly, these estimates were 5.44 vs. 4.02 for LDL cholesterol; 5.94 vs. 2.74 for HDL cholesterol; 2.07 vs. 1.40 for VLDL cholesterol; and 10.13 vs. 6.31 for triglycerides. Notably, females were responsible for both the low and the high value in the distributions of all lipids. In particular, standardized variance in total cholesterol levels among females was more than double that for males. This finding appears to be primarily due to the large differences in variance between males and females for HDL cholesterol specifically, as standardized variance in HDL cholesterol levels for females is also more than twice that for males.

**Table 7:** Additive genetic effects on spontaneous lipid levels in 193 pedigreed rhesus macaques fed a standard chow diet low in fat and cholesterol.

**TRAIT**	**h^2^**	**SEM**	**P-value**	**Significant covariates**	**c^2^**
**TCHOL**: (N=193)	**0.257**	0.188	**0.032**	Sex, Age × Sex, Age^2^ × Sex	0.169
FEMALES (N=125)	**0.644**	0.267	**0.004**	Age, Age^2^	0.160
MALES (N=68)	0.000	N/A	0.500	-----	----
**LDL-C**: (N=193)	**0.252**	0.176	**0.030**	Age, Age^2^	0.188
FEMALES (N=125)	0.225	0.215	0.075	Age, Age^2^	0.180
MALES (N=68)	1.000	N/A	0.057	Age, Age^2^	0.135
**HDL-C**: (N=193)	0.227	0.216	0.103	Sex, Age × Sex, Age^2^ × Sex	0.105
FEMALES (N=125)	**0.843**	0.216	**0.0008**	Age, Age^2^	0.076
MALES (N=68)	0.000	N/A	0.500	None	N/A
**VLDL-C**: (N=193)	0.166	0.135	0.057	Sex	0.025
FEMALES (N=125)	0.000	N/A	0.500	Age	0.132
MALES (N=68)	**0.482**	0.299	**0.018**	None	N/A
**TRIG**: (N=193)	**0.197**	0.139	**0.034**	None	N/A
FEMALES (N=125)	0.0002	0.151	0.499	Age	0.141
MALES (N=68)	**0.705**	0.293	**0.001**	None	N/A

**TCHOL**: total cholesterol; **LDL-C**: LDL cholesterol; **HDL-C**: HDL cholesterol; **VLDL-C**: VLDL cholesterol; **TRIG**: triglycerides; **h^2^**: proportion of residual phenotypic variance (phenotypic variance remaining after accounting for covariate effects) due to additive genetic effects; **SEM**: standard error; **c^2^**: proportion of observed phenotypic variance due to measured covariates. All genetic analyses conducted using SOLAR© (1995-2004) software.

**Figure 3:**
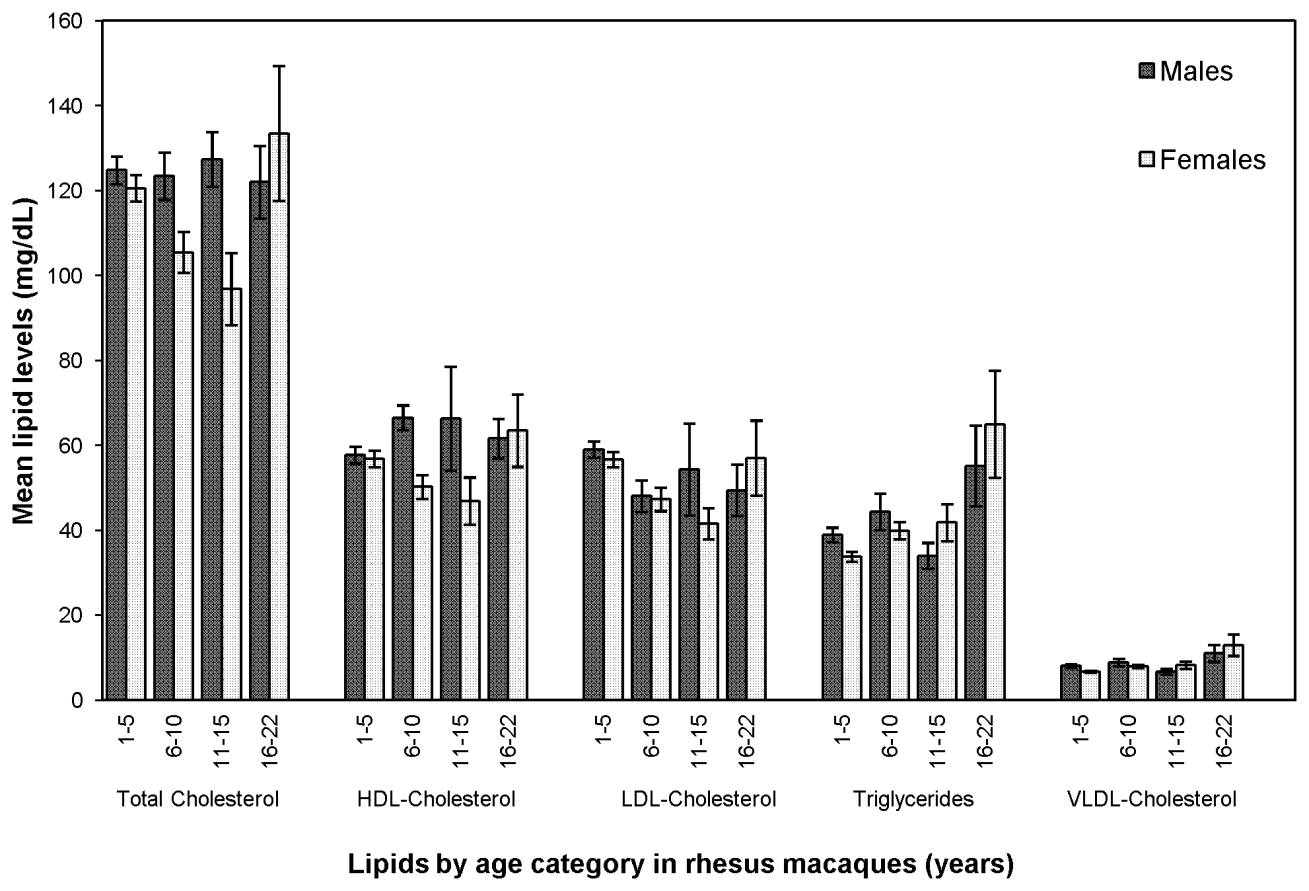
Mean lipid levels across age categories for 125 female and 68 male pedigreed rhesus macaques.

In addition to effects of sex, interaction between age and sex is also suggested by an examination of trends in observed lipid levels across age categories, and indeed has statistically significant effects on some (TCHOL and HDL-C, TABLE **7**), although not all, lipids. Differences between males and females appear to influence mean VLDL-C and triglyceride levels in particular for the oldest animals (age 16-22 years, corresponding to 48-66 human years, see Figure **3**). When examined across approximately even age categories and sex, mean levels of all lipids in males are roughly equivalent over all age categories (1-5 years, N=39; 6-10 years, N=19; 11-15 years, N=3; and 16-22 years, N=7), with the exception of VLDL-C and triglycerides. In females, while mean levels of total, LDL, and HDL cholesterol are consistently decreased across the first 3 age categories (1-5 years, N=63; 6-10 years, N=38; and 11-15 years, N=17), mean levels of VLDL cholesterol and triglycerides are increased across these same age categories. Strikingly, for both males and females in the oldest age category (16-22 years, corresponding to 48-66 years in humans; N=7 each for both males and females), mean levels for VLDL-C and triglycerides are increased substantially compared to previous age categories. Comparisons to the weighted means of all previous age categories indicate that, for the oldest males, VLDL-C and triglyceride levels are increased by ~33% and 37%, respectively; for the oldest females, mean total and HDL-C levels are increased by ~19%, while VLDL-C and triglyceride levels are increased by ~76% (data not shown). In addition, females in the oldest age category have greater mean levels of all lipids investigated than the same-aged males.

### Additive genetic effects on lipid levels

When all lipid measures were evaluated in the full sample (N=193), we detected moderate but significant additive genetic contributions to total cholesterol, LDL-C, and triglyceride levels only (P=0.030–0.034), summarized in TABLE 7), with these contributions explaining ~20–26% of residual phenotypic variance after accounting for significant covariates related to sex, age, and their interaction. The estimated heritability for VLDL-C levels was lower at ~0.17, and this estimate borders on statistical significance (P=0.057). Unexpectedly, genetic contributions to HDL-C levels could not be reliably described in the full sample (*h* ^2^ ~ 0.23, P = 0.103).

With the sole exception of LDL-C, when the sample was stratified by sex, substantial sex-specific heritability was either revealed for the first time (HDL-C, VLDL-C) or was increased greatly over what had been found in the pooled sample (total cholesterol, triglycerides). In HDL-C levels, additive genetic effects explained ~84% (P=0.0008) of residual phenotypic (co) variance in females, while significant heritability was not detected in males. Conversely, additive genetic effects explained ~48% (P=0.018) of co-variation in VLDL-C levels in males, but significant heritability was not detected in females. For total cholesterol levels, heritability increased to 0.644 (P = 0.004) in females, but was not detected in males, while for triglycerides, heritability increased to 0.705 (P = 0.001) in males, but was not detected in females. For LDL-C levels, estimates of sex-specific heritability were marginally significant in both sexes, with complete heritability indicated for males (*h* ^2^ = 1.000, P = 0.057) and a more moderate heritability detected for females (*h* ^2^ = 0.225, P = 0.075).

## Discussion

The rhesus macaque is a well-established model for the influence of dietary cholesterol on the initiation and progression of atherosclerosis. Rhesus macaques develop both mild and more extreme hypercholesterolemia (primarily in LDL) that correlates well with the severity and duration of experimental cholesterol feeding [2,3,5,7-9], and with the appearance of fatty streaks and more complicated lesions in the aorta and coronary arteries [5-8]. Under extended experimental diets, rhesus macaques also display pathological and clinical sequelae that are similar to those observed in humans, including coronary artery stenosis, lesion mineralization, thrombosis, myocardial infarction, and sudden death [1,5]. Surprisingly, despite this body of research and the substantial genetic similarity between rhesus macaque and human, this species has not been used as a model for genetic discovery in human atherosclerosis. This study took a first step toward this goal by investigating the genetic contribution to spontaneous lipid levels in a sample of Indian-origin rhesus macaques selected from a single, extended pedigree developed for statistical power in quantitative trait analysis.

Levels of all lipids investigated in this study were characterized by remarkable variation, a finding similar to a previous report of variation in lipids in older macaques with spontaneous hypercholesterolemia [29]. In particular, we observed total cholesterol, triglyceride, and VLDL-C levels at or beyond 3 standard deviations from their sex-specific means in both males and females (corresponding to >99^th^ percentile for normally distributed data). These results are striking because these traits were measured in healthy, unchallenged animals maintained on a diet low in fat and cholesterol compared to an average Western diet. Considering the substantial dietary and other environmental homogeneity experienced by these animals, the magnitude of this variation alone suggests significant heritability for spontaneous lipid levels, and implies that macaques are also likely to vary in genetic susceptibility to atherosclerosis, consistent with observations in humans.

In agreement with this idea, we found a moderate but significant additive genetic contribution accounting for ~20-26% of observed variation in total cholesterol, LDL cholesterol, and triglyceride levels in the full sample of 193 macaques after adjustment for covariates of age and sex. These results are consistent with age- and sex-adjusted heritability estimates of 17-28% for these lipids reported in a similar study of 868 men and women in an isolated population characterized at random with respect to dyslipidemia [the Erasmus Rucphen Family Study; 30]. However, higher estimates of heritability for circulating lipids have been reported in many other human studies [e.g., 31-32], and the apparent lack of significant heritability for HDL cholesterol in particular in our full sample was unexpected. Sample stratification by sex revealed the source of these anomalies to be heterogeneity due to strong sex-specific additive genetic effects. When the sample was stratified by sex, substantial heritability was either revealed for the first time (e.g., for HDL cholesterol in female macaques and VLDL cholesterol in male macaques), or was increased 2- to 3-fold over heritability assessed with males and females combined (e.g., for total cholesterol in females, LDL cholesterol in males, and triglycerides in males). We note that, although the estimate of sex-specific heritability for LDL cholesterol levels appears to be complete in males (i.e., h^2^ = 1.0) and moderate in females, these estimates only achieved marginal statistical significance, and complete heritability of LDL cholesterol in males in particular is unlikely. Rather, the small number of males in this study likely prevents an accurate estimate of sex-specific heritability for this lipid species. In general, a limitation of our sex-stratified analyses is the small number of males relative to the number of females. Although the small number of males used in these analyses was sufficient to reveal substantial heritability for VLDL cholesterol and triglyceride levels, the heritability of total and HDL cholesterol levels found only in females in this study may also extend to males once larger numbers of males are investigated in a more balanced experimental design. Despite these limitations, our estimates of sex-specific heritability for total cholesterol, HDL cholesterol, VLDL cholesterol, and triglycerides (range 0.482–0.843) in 68 male or 125 female rhesus macaques are either greater than, or equivalent to, age- and sex-adjusted heritability estimates reported for these lipids in human studies based on 7- to 8-fold larger sample sizes that include both men and women [31,32].

Accumulating evidence indicates that genetic influences on lipids and lipid metabolism differ by sex, and that accounting for sex-specific genetic architecture may be critical to identifying susceptibility loci for lipid traits [17-19]. Of particular relevance to this study, Weiss et al. [17] found that heritability for LDL and HDL cholesterol levels differed significantly between Hutterite men and women, with HDL cholesterol levels showing evidence for sex-specific linkage that did not occur in the combined sample. Genetic determinants of lipid metabolism may also be sex-specific, and may differ from genetic effects on lipid levels, as evidenced by a recent study describing sex-specific genetic variants in *LIPC*, *ABCA1*, *APOA1*, and *APOAII* associated with differences in the capacity of plasma to mediate cholesterol efflux from human macrophages independent of circulating HDL cholesterol levels [19]. In comparison, a recent study of the transcriptome in liver tissue from men and women identified over 1,200 genes that displayed sex-biased expression, including *LIPC*, *ABCA1*, *LDLR*, and *APOA5*, which have been previously associated with dyslipidemia, including monogenic forms. All of these genes showed sex-specific patterns of expression consistent with known differences between men and women in lipid profiles and related cardiovascular risk [18]. Despite these seeming inconsistencies, our own findings of sex-specific heritability for total cholesterol, HDL-C, VLDL-C, and triglycerides in rhesus macaques suggest potential differences between male and female macaques in genetic variation regulating lipid levels, which may extend to differences in genetic variation regulating lipid metabolism, in the context of a standard chow diet.

This is the first study to demonstrate the heritability of cholesterol levels in rhesus macaques. Although variation in baseline lipids and in response to a high-fat/high-cholesterol diet has been observed previously in this species, previous inference of genetic effects on this variation has been limited to observed differences among macaque and other monkey species in response to the same experimental diet [2,9]. Whether genetic variation that influences baseline cholesterol levels in response to a low-fat, low-cholesterol diet is the same as that influencing cholesterol levels in response to an experimental diet is currently an unanswered question, but it would be surprising if these gene sets were mutually exclusive. Thus, the genetic effects we have detected in this study may also influence response to increased dietary fat and cholesterol, and by extension the more widespread and complicated atherosclerosis in macaques that accompanies elevated lipid levels in the periphery.

In addition to demonstrating significant heritability for all lipid levels (exclusive of the effects of age, sex, and their interaction, as indicated), we observed differences in the distribution of lipid levels between male and female rhesus macaques for all lipids that suggest significant sexual dimorphism in these traits. These results are consistent with the significance of sex effects observed for several lipids in this study, and the sexual dimorphism observed in humans for these and other cardiovascular risk factors [33,34]. While male macaques had higher mean and median values for all lipids than did females, females had greater standardized variance than males for all lipids (ranging from 35% greater for LDL-C to 117% greater for HDL-C). Of note, 8 females (but no males) had lipid profiles that combined HDL-C levels at or below the 15^th^ percentile with triglyceride levels at or above the 85^th^ percentile of their sex-specific distributions, with 2 of these 8 females having even more extreme HDL-C and triglyceride levels in the 5^th^ and 95^th^ percentiles, respectively. This dyslipidemic profile has been associated previously with metabolic syndrome and type 2 diabetes in rhesus macaques [35], suggesting the potential of this species to elucidate genetic contributions to cardiovascular risk in these disorders.

Significant interaction between sex and age is also suggested by the considerable increase observed in mean total cholesterol, VLDL-C, and triglyceride levels in the oldest males and females compared to younger macaques of the same sex, and because this increase in mean VLDL-C and triglyceride levels in the oldest females is more than twice that of the oldest males. These observations reflect similar trends in human populations, in which gender, age, and their interaction are important predictors of risk for coronary heart disease. Similar to our observations in female macaques, triglyceride levels rise steadily over a woman’s lifetime, but increase faster in women after age 50 [36,37]. Increased triglycerides have been independently associated with significantly greater risk for adverse CVD events, but substantially more so for women than for men [38], in whom triglyceride levels are greater and rise faster than women until the age of 50 but plateau thereafter [37]. Also similar to our findings in female macaques, in addition to faster increases in triglyceride levels after age 50, women also have faster increases in total cholesterol at the same timepoint, and often have higher total cholesterol than men of the same age [33,34,37]. As the oldest age category for female macaques in this study spans the age at which reproduction ceases naturally, our observations of substantial increases in mean total cholesterol, VLDL-C, and triglyceride levels in the oldest female macaques may mirror similar trends in middle-aged and postmenopausal women that result in increased cardiovascular risk [33]. However, the absence of heritability for VLDL-C and triglyceride levels in female macaques suggests that our observations of increases in these lipids in the oldest females may be due to influences on these traits other than additive genetic effects, such as epistatic, gene-by-environment interaction, or specific environmental effects not modeled in our analyses.

In addition to the similarities noted above, we also observed differences in some age-related trends in lipid levels between macaques in this study and longitudinal studies conducted in humans. Although smaller in magnitude compared to similar findings in female macaques, substantial increases in mean VLDL-C and triglycerides observed in the oldest male macaques compared to younger males are intriguing, as these lipids are expected to plateau or decline in men of corresponding age. Other differences observed include the increase in mean HDL-C levels for the oldest female macaques in contrast to expected decreases in middle-aged women, and the relative stability of mean LDL-C levels over all age categories for both sexes, in contrast to the increases in LDL-C usually observed in middle-aged men and women. These observed discrepancies in LDL-C and HDL-C trends over age between macaques and humans are likely due to dietary differences between the low-fat, low-cholesterol diet fed to macaques in this study, and the average Western diet likely consumed by most human research participants; however, confirmation of this idea will need to be addressed in future research.

The close evolutionary relationship between macaques and humans, combined with the ability to control diet and other environmental variables, suggest that future investigation in the genetic determinants of lipid levels in macaques is likely to reveal both known and novel results that may inform human health. Genetic influences on lipid levels in humans are well-documented [10,11], and our findings of heritability for lipid levels in macaques is consistent with these reports, and with the high degree of genetic similarity between the two species. This similarity is exemplified by published reports of familial disorders of lipid metabolism in both humans and macaques associated with mutations in *LDLR* [39], and by the corresponding degree of homology found between the two species for this gene. The rhesus macaque *LDLR* exhibits from 95–100% identity with the human *LDLR* and its corresponding LDL receptor protein at multiple levels of organization, including gene length, open reading frame length, protein length, and mRNA transcript sequence identity, and from 85–100% identity with the human *LDLR* across functional domains in the protein [40,41]. This degree of genetic similarity between macaques and humans in *LDLR* is consistent with phenotypic similarity between humans and macaques that fulfill (human) clinical criteria for familial hypercholesterolemia [42], including elevated total and LDL cholesterol levels and reduced LDL receptor expression.

In this study, we present a first-ever report of total and sex-specific heritability for circulating levels of total cholesterol, LDL cholesterol, HDL cholesterol, VLDL cholesterol, and triglycerides in a relatively small number of pedigreed rhesus macaques. Our results indicate that the Indian-origin rhesus macaque is likely to be a highly efficient model for discovery of sex-specific genetic determinants of lipid traits known to influence human atherosclerosis. Moreover, because our findings demonstrate a sex-specific and substantial genetic component to baseline lipid levels in a low-fat chow diet, they also suggest that the rhesus macaque may be a valuable model for sex-specific genetic effects on elevated cholesterol levels in response to experimental diet, on the extent and severity of atherosclerosis that accompanies elevated cholesterol levels, and on the clinical sequelae observed in this species. Finally, the results described in this study confirm the utility of the pedigree and associated sample resources developed for the purpose of this and future studies, and underscore the need to discover genetic variants influencing spontaneous lipid levels in this population.
