# Correction: Sex-Specific Heritability of Spontaneous Lipid Levels in an Extended Pedigree of Indian-Origin Rhesus Macaques (*Macaca mulatta*)

**DOI:** 10.1371/annotation/1dc8bed5-aef7-4251-a23d-bded0b0ee9a4

**Published:** 2014-01-09

**Authors:** Amanda Vinson, Asia D. Mitchell, David Toffey, Jacob Silver, Michael J. Raboin

There were errors introduced during the preparation of this manuscript for publication. Some symbols in the “Methods” section under the sub-heading “Quantitative Genetic Analysis” were deleted. The correct text, including the symbols, for this sub-heading can be found here:

http://plosone.org/corrections/pone.0072241.000.cn.tif

